# Psychiatric disorders in patients with benign and malignant sinonasal tumors: a prospective cross-sectional study

**DOI:** 10.3389/fpsyg.2024.1444522

**Published:** 2024-12-11

**Authors:** Guang-Ye Zhan, Hui-Fang Wang, Dong-Fang Wang, Yi-Hui Wen, Hua Zhong, Wei-Ping Wen, Jian Li, Liang Peng

**Affiliations:** ^1^Department of Otorhinolaryngology Head and Neck Surgery, The First Affiliated Hospital, Sun Yat-sen University, Guangzhou, Guangdong, China; ^2^Institute of Otorhinolaryngology Head and Neck Surgery, Sun Yat-sen University, Guangzhou, Guangdong, China; ^3^Department of Otorhinolaryngology, SanMing First Hospital, Fujian Medical University, Sanming, Fujian, China; ^4^Department of Otorhinolaryngology, Guangxi Hospital Division of the First Affiliated Hospital, Sun Yat-sen University, Nanning, Guangxi, China

**Keywords:** depression, anxiety, insomnia, somatic symptom disorder, sinonasal tumor

## Abstract

**Objective:**

To assess the prevalence of depression, anxiety, insomnia, and somatic symptom disorder (SSD) in patients with benign and malignant sinonasal tumors.

**Materials and methods:**

Pretreatment patients with sinonasal tumors were prospectively recruited on the rhinology ward of a tertiary hospital from July 2021 to March 2022. The electronic questionnaire which contains the rhinological symptom scale, the 22-item Sinonasal Outcome Test (SNOT-22) Scale, the Patient Health Questionnaire-9 (PHQ-9), the Generalized Anxiety Disorder-7 (GAD-7), the Insomnia Severity Index (ISI), and the Patient Health Questionnaire-15 (PHQ-15) was filled out by patients at admission. The associations between the scores of symptom/SNOT-22 and psychometric tests were assessed by the Pearson correlation coefficient (*r*) and simple linear regression. The receiver operating characteristic (ROC) analysis was used to evaluate the performance of the SNOT-22 score in predicting psychiatric disorders.

**Results:**

Thirteen patients with benign sinonasal tumors and 15 patients with malignant sinonasal tumors were recruited. The benign and malignant groups did not differ significantly regarding symptomatology and mental wellbeing. Of the total patients, 9 were at risk of depression (PHQ-9 > 4), 10 were at risk of anxiety (GAD-7 > 4), 11 were at risk of insomnia (ISI > 7), and 11 were at risk of SSD (PHQ-15 > 4). The overall symptom, facial pain/pressure, postnasal drip, and SNOT-22 scores were positively associated with scores of psychometric tests. Patients with a high SNOT-22 score (>18) are likely to be affected by comorbid psychiatric disorders. When interpreting the results of this study, it should be noted that screening tools, not diagnostic tools, were used to identify psychiatric risk.

**Conclusion:**

Depression, anxiety, insomnia, and SSD are prevalent in patients with sinonasal tumors. Otolaryngologists should have a low threshold to ask the patient about psychiatric symptoms, especially for patients with an SNOT-22 score > 18.

## 1 Introduction

Sinonasal tumors are a wide spectrum of benign and malignant tumors with various tissues of origin (Nagornaya et al., 2023). The diagnosis of sinonasal tumor may be delayed significantly due to the overlap of presenting symptoms with inflammatory sinonasal diseases. In addition to nasal symptoms, patients with sinonasal tumors may suffer from ocular and intracranial complications as well as disfigurement. In particular, patients with sinonasal tumors may experience unique challenges associated with visual changes, anosmia, the social stigma of facial disfiguration, and pain associated with nasal obstruction (Lee et al., 2020). Patients’ quality of life (QOL) is usually significantly impaired by sinonasal tumors before and after treatments (Fleseriu et al., 2023; Philips et al., 2021). Although there is a lack of standard QOL instruments for sinonasal tumors, the 22-item Sinonasal Outcome Test (SNOT-22) has become one of the common tools used in sinonasal tumors in previous studies (Glicksman et al., 2018; Shah et al., 2020; Chow et al., 2022).

Mental health is an important dimension of QOL. It has been suggested that QOL is lowered in patients with sinonasal tumors due to the significant psychological stress (James Fasunla and Omokanye, 2011). In addition to the symptomatology, socioeconomic, and financial burden associated with tumors, the diagnosis of sinonasal tumor itself is a psychological stressor (Derogatis et al., 1983). However, mental health has been poorly assessed in literature, either using generic QOL tools or excluding sinonasal benign tumors (Philips et al., 2021; Bhenswala et al., 2019). There is an urgent need for further studies screening for psychiatric disorders in patients with sinonasal tumors.

Here, we conducted a prospective cross-sectional study and aimed to screen for psychiatric disorders, including depression, anxiety, insomnia, and somatic symptom disorder (SSD), in patients with sinonasal tumors before treatment. Moreover, we tried to explore the relationship between symptomatology and psychiatric disorders and the difference between benign and malignant tumors.

## 2 Materials and methods

### 2.1 Setting

The rhinology ward at our institution, a tertiary hospital.

### 2.2 Participants

Adult patients (>18 years) diagnosed with benign or malignant sinonasal tumors, who were admitted to the rhinology ward for further workup and treatment and were able to understand and complete the study questionnaires, were included. Patients with preexisting psychiatric diagnoses were excluded. A total of 28 inpatients (13 with benign tumors and 15 with malignant tumors) were prospectively recruited from July 2021 to March 2022. At admission, patients were asked to fill out an electronic questionnaire, which contains the Rhinological Symptom Scale, SNOT-22 scale, and four specific screening scales for psychiatric disorders, and patients’ demographic and socioeconomic characteristics were also collected. The subsequent treatment plan for each patient was not influenced by this study. This study was approved by the Institutional Review Board of our institution. All participants signed the electronic informed consent.

### 2.3 Symptomatology assessment

The common rhinological symptoms over the last 2 weeks were scaled from 0 to 10, with higher values indicating more severe symptoms. The SNOT-22 scale was also used to evaluate patients’ symptomatology and QOL.

### 2.4 Screening of psychiatric disorders

The Patient Health Questionnaire-9 (PHQ-9) was used to screen for depression, with a sensitivity of 88% and a specificity of 85% (Kroenke et al., 2001; Levis et al., 2019). The PHQ-9 scores range from 0 to 27, and total scores of 5, 10, 15, and 20 represent cut points for mild, moderate, moderately severe, and severe depression, respectively. Patients with a PHQ-9 score > 4 were considered at risk of depression. The Generalized Anxiety Disorder-7 (GAD-7) was used to screen for generalized anxiety disorder (GAD), with a sensitivity of 89% and a specificity of 82% (Spitzer et al., 2006). The GAD-7 scores range from 0 to 21, and total scores of 5, 10, and 15 represent cut points for mild, moderate, and severe anxiety, respectively. Patients with a GAD-7 score > 4 were considered at risk of GAD. The Insomnia Severity Index (ISI) was used to screen for insomnia, with a sensitivity of 86% and a specificity of 88% (Bastien et al., 2001; Morin et al., 2011). The ISI scores range from 0 to 28, and total scores of 8, 15, and 23 represent cut points for subthreshold, moderate, and severe insomnia, respectively. Patients with an ISI score > 7 were considered at risk of insomnia. The Patient Health Questionnaire-15 (PHQ-15) was used to screen for SSD, with a sensitivity of 78% and a specificity of 71% (Kroenke et al., 2002; van Ravesteijn et al., 2009). The PHQ-15 scores range from 0 to 30, and total scores of 5, 10, and 15 represent cut points for low, medium, and high somatic symptom severity, respectively. Patients with a PHQ-15 score > 4 were considered at risk of SSD.

### 2.5 Statistical analyses

Characteristics were compared between patients with benign tumors and those with malignant tumors using Pearson’s chi-squared test or Fisher’s exact test or independent-samples *t*-test as appropriate. The symptom scores, SNOT-22 scores, and scores of psychometric tests were compared between the benign and malignant groups using Mann–Whitney *U*-test. The associations between symptom/SNOT-22 scores and scores of psychometric tests were assessed by the Pearson correlation coefficient (*r*) and simple linear regression. Furthermore, the receiver operating characteristic (ROC) analysis was used to assess the performance of the SNOT-22 score in predicting psychiatric disorders. Statistical analyses were conducted using IBM SPSS Statistics version 26. A two-sided *p*-value ≤0.05 was considered statistically significant.

## 3 Results

Of the 13 recruited patients with benign sinonasal tumors, six were diagnosed with inverted papilloma, four with ossifying fibroma, one with neurilemmoma, one with pleomorphic adenoma, and one with juvenile nasopharyngeal angiofibroma. Of the 15 recruited patients with malignant sinonasal tumors, five were diagnosed with squamous cell carcinoma, three with olfactory neuroblastoma, two with sarcoma, two with adenocarcinoma, one with adenoid cystic carcinoma, one with melanoma, and one with plasmacytoma. The small sample size and heterogeneous histologic types of sinonasal tumors should be considered when interpreting the results of our study. Patients of the benign tumor group were younger than those of the malignant tumor group. No significant differences were detected in other demographic or socioeconomic variables between the two groups (Table 1). Of the total 28 patients with sinonasal tumors, there were 9 (32.1%) with PHQ-9 scores >4, 10 (35.7%) with GAD-7 scores >4, 11 (39.3%) with ISI scores >7, and 11 (39.3%) with PHQ-15 scores >4. The prevalence rates of psychiatric disorders were similar between the benign and malignant groups (Table 1).

**Table 1:** Characteristics of participants.

Characteristics	Benign tumor	Malignant tumor	*p*-value
Sex			0.743
Male	7 (53.8)	9 (60.0)	
Female	6 (46.2)	6 (40.0)	
Educational attainment			0.274
Below college diploma	6 (46.2)	10 (66.7)	
College diploma or above	7 (53.8)	5 (33.3)	
Family income per year			
<80,000 CNY	10 (76.9)	9 (60.0)	0.435
≥80,000 CNY	3 (23.1)	6 (40.0)	
Marriage			0.198
Married	8 (61.5)	13 (86.7)	
Unmarried/Others	5 (38.5)	2 (13.3)	
Insurance			1.000
Yes	12 (92.3)	13 (86.7)	
No	1 (7.7)	2 (13.3)	
Age (year)	32.4 ± 9.2	45.9 ± 13.2	0.005
Body mass index (kg/m^2^)	23.0 ± 6.2	24.5 ± 6.0	0.541
PHQ-9 score			1.000
≤4	9 (69.2)	10 (66.7)	
>4	4 (30.8)	5 (33.3)	
GAD-7 score			1.000
≤4	8 (61.5)	10 (66.7)	
>4	5 (38.5)	5 (33.3)	
ISI score			0.934
≤7	8 (61.5)	9 (60.0)	
>7	5 (38.5)	6 (40.0)	
PHQ-15 score			0.488
≤4	7 (53.8)	10 (66.7)	
>4	6 (46.2)	5 (33.3)	

Data are presented as number (%) or mean ± standard deviation. *p*-values are calculated by Pearson’s chi-squared test or Fisher’s exact test for categorical variables as appropriate and independent-samples *t*-test for continuous variables. PHQ-9, Patient Health Questionnaire-9; GAD-7, Generalized Anxiety Disorder-7; ISI, Insomnia Severity Index; PHQ-15, Patient Health Questionnaire-15.

**Figure 1:**
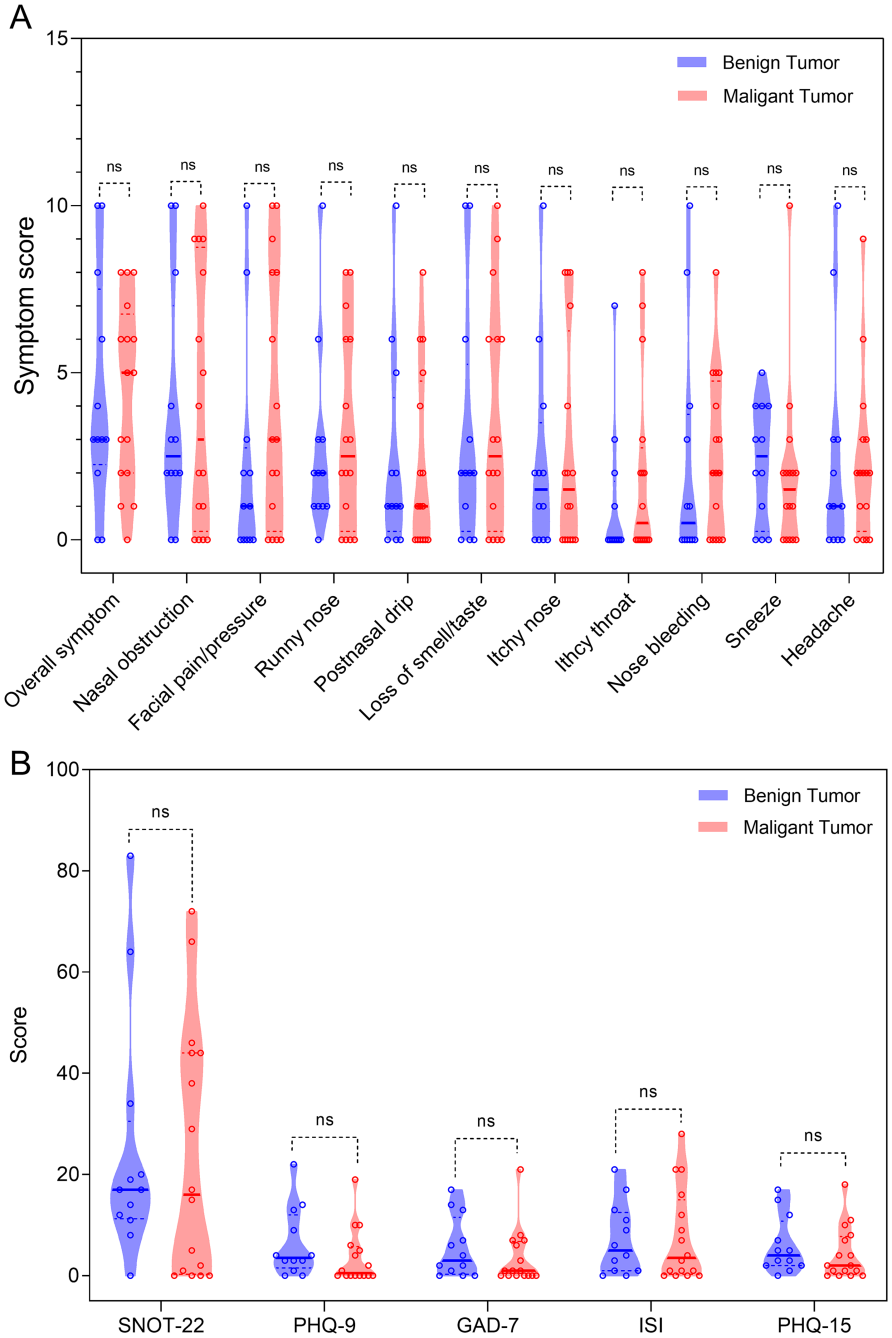
Violin plots presenting the comparisons between the benign and malignant groups. **(A)** The rhinological symptom scores; **(B)** the SNOT-22 score and scores of psychometric tests. Mann–Whitney *U*-test was used. ns, not significant; PHQ-9, Patient Health Questionnaire-9; GAD-7, Generalized Anxiety Disorder-7; ISI, Insomnia Severity Index; PHQ-15, Patient Health Questionnaire-15; SNOT-22, 22-item Sinonasal Outcome Test.

**Figure 2:**
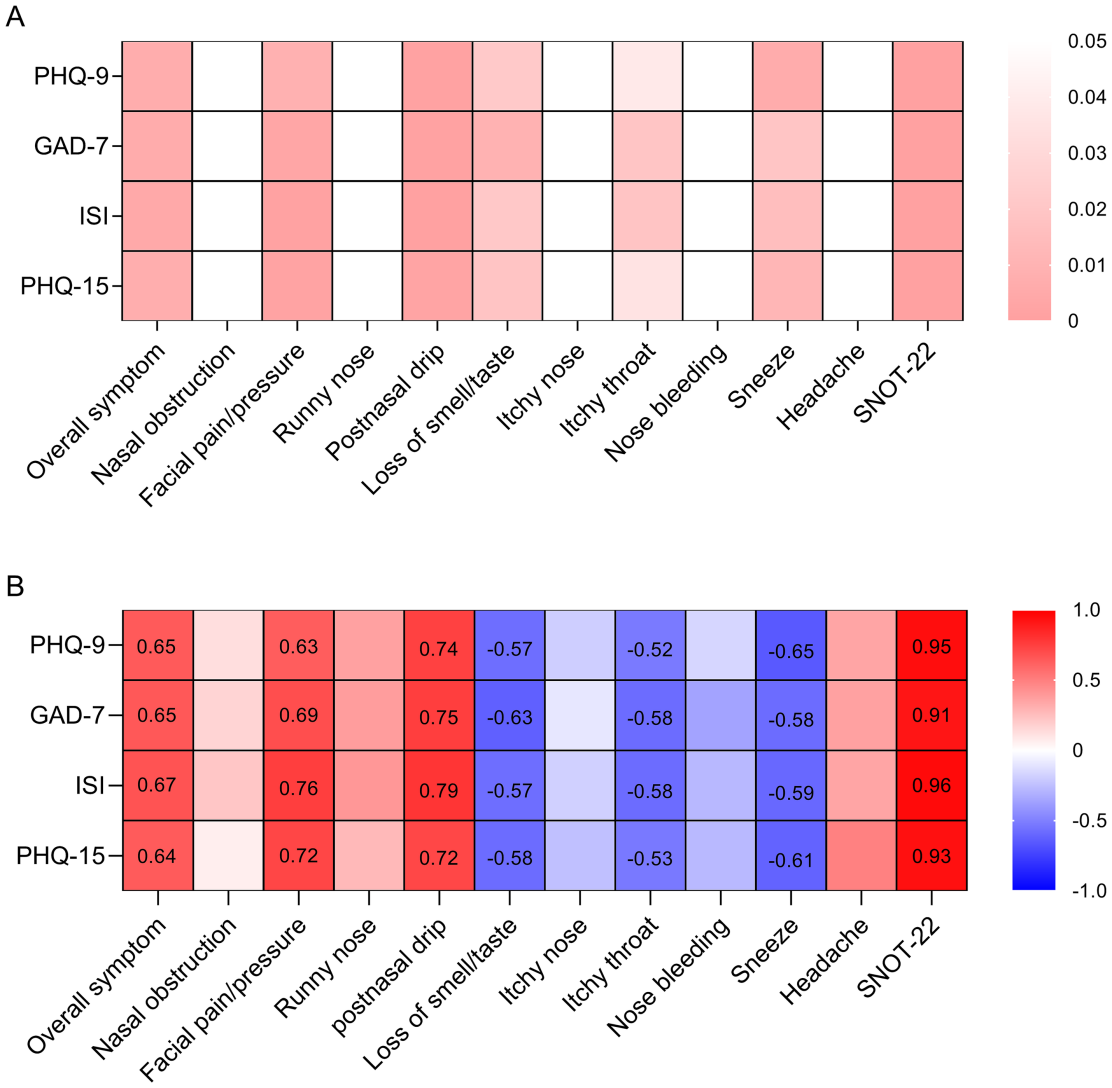
Hot plots presenting the correlations between symptom/SNOT-22 scores and scores of psychometric tests in the whole cohort. **(A)** The *p*-values of Pearson correlation; **(B)** the Pearson correlation coefficients. PHQ-9, Patient Health Questionnaire-9; GAD-7, Generalized Anxiety Disorder-7; ISI, Insomnia Severity Index; PHQ-15, Patient Health Questionnaire-15; SNOT-22, 22-item Sinonasal Outcome Test.

**Figure 3:**
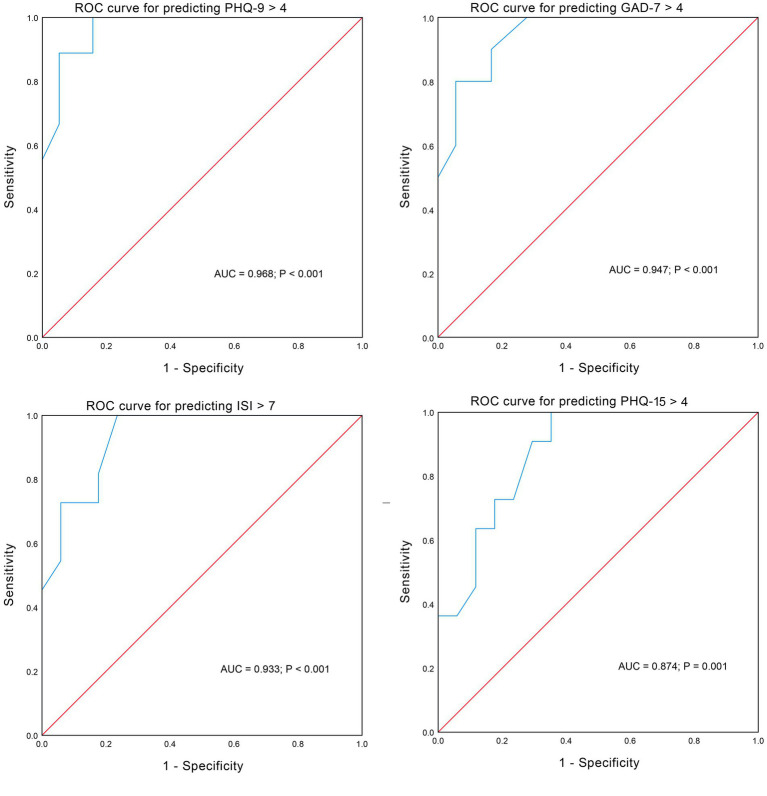
Scatter plots with linear fitting curves presenting the correlations between symptom/SNOT-22 scores and scores of psychometric tests in benign and malignant groups separately. PHQ-9, Patient Health Questionnaire-9; GAD-7, Generalized Anxiety Disorder-7; ISI, Insomnia Severity Index; PHQ-15, Patient Health Questionnaire-15; SNOT-22, 22-item Sinonasal Outcome Test.

The rhinological symptom scores, SNOT-22 scores, and scores of psychometric tests were comparable between the benign and malignant groups (Figure 1). For the whole cohort, the overall symptom, facial pain/pressure, postnasal drip, and SNOT-22 scores were positively associated with scores of psychometric tests, whereas the loss of smell/taste, itchy throat, and sneeze scores were negatively associated with scores of psychometric tests (Figure 2). We further analyzed the correlations in the benign and malignant groups separately (Figure 3). The SNOT-22, overall symptom, facial pain/pressure, and postnasal drip scores remained positively associated with scores of psychometric tests in the benign and malignant groups. However, the negative associations did not exist in the benign or malignant group, which means that the negative correlation in the whole group is a statistical fallacy.

**Figure 4:**
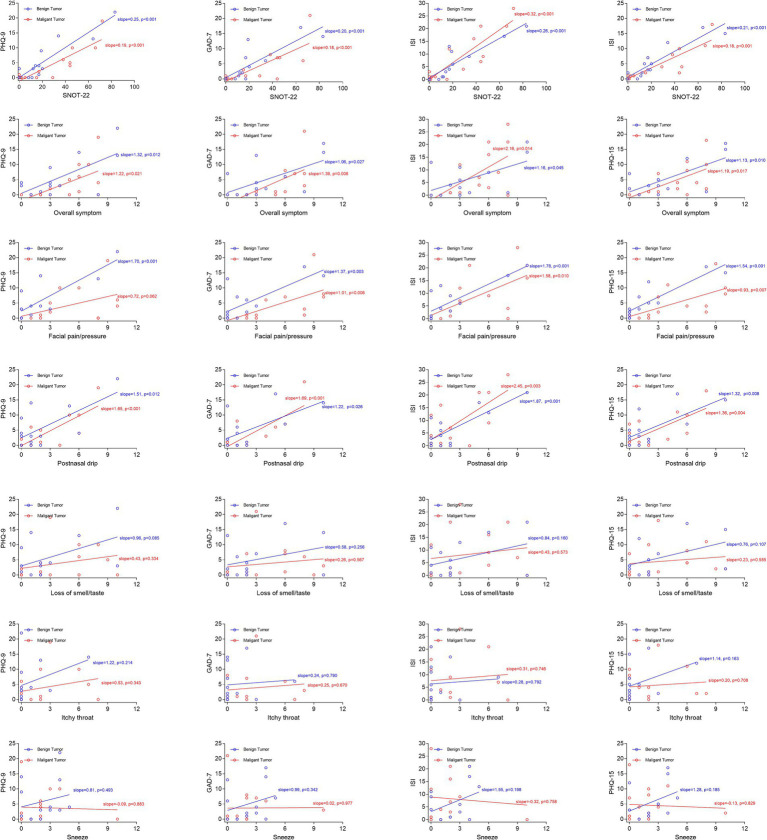
Receiver operating characteristic curves showing the performance of the SNOT-22 score in predicting psychiatric disorders.

The SNOT-22 score was most closely correlated with scores of psychometric tests (*r* > 0.9). ROC analyses confirmed the good discrimination performance of psychiatric disorders prediction by the SNOT-22 score, with an area under the curve (AUC) all larger than 0.85 (Figure 4). We found that a score of 18 was a relatively good cutoff value for the SNOT-22 which allowed good sensitivity and specificity in predicting psychiatric disorders. The sensitivity and specificity were 100 and 84.2% for predicting PHQ-9 > 4, 90.0 and 83.3% for predicting GAD-7 > 4, 81.8 and 82.4% for predicting ISI > 7, and 72.7 and 76.5% for predicting PHQ-15 > 4.

## 4 Discussion

The topic of psychiatric assessments in oncology settings is an active area of research in literature (Wang et al., 2024; Tao et al., 2024; McGrady et al., 2024; Annunziata et al., 2020; Haywood et al., 2024; Oberoi et al., 2024; Lan et al., 2024). To the best of our knowledge, this study is the first to screen for psychiatric disorders including depression, anxiety, insomnia, and SSD in patients with sinonasal tumors. It is noted that the scores of psychometric tests should not be regarded as the definitive diagnostic criteria of psychiatric disorders, and hence, the prevalence rates of psychiatric disorders in patients with sinonasal tumors reported in our study may be biased from the true rates to some extent. The correct interpretation of screening results should be the proportion of patients at risk of psychiatric disorders. Considering the above, we adopted the minimal criteria of these screening tools for defining patients at risk of psychiatric disorders.

Literature on mental health in patients with sinonasal tumors is very limited. A large retrospective cohort study reported that the prevalence rates of mental health disorders, which included depression, anxiety, and adjustment disorders, were 22 and 31% before and after the diagnoses of sinonasal cancer (Lee et al., 2020). Another retrospective study reported that 34.6% of patients with sinonasal malignancies who received definitive treatment were at risk of depression or anxiety based on the Hospital Anxiety and Depression Scale (Philips et al., 2021). No data about insomnia or SSD has been reported in patients with sinonasal tumors in previous studies. In the current study, we reported a 32.1% prevalence rate of depression (based on PHQ-9 > 4), a 35.7% prevalence rate of anxiety (based on GAD-7 > 4), a 39.3% prevalence rate of insomnia (based on ISI > 7), and a 39.3% prevalence rate of SSD in patients with sinonasal tumors. The prevalence rates of psychiatric disorders were higher in patients with sinonasal tumors than those in the general population (Shin et al., 2020; Löwe et al., 2008; Ohayon, 2002; Kocalevent et al., 2013). Patients’ mental wellbeing is an important goal of treatment. Identifying patients at risk of psychiatric disorders enables a multidisciplinary team to be more responsive to patient’s needs and concerns extending into effective pretreatment counseling (Deshpande et al., 2011).

The malignant tumor itself is an unneglectable psychiatric stressor, which could lead to psychological consequences. Even though the tumor is benign, the mass can still cause severe symptoms and impair the QOL. For example, the ossifying fibroma may disfigure the patient’s face and result in decreased socializing and increased risk of psychiatric disorders. In the current study with a pretreatment setting, compared with patients with malignant sinonasal tumors, patients with benign sinonasal tumors had similar symptom scores, SNOT-22 scores, and scores of psychometric tests. Our results are consistent with the findings of Krampe et al. that surgical patients with various malignant tumors and those with benign tumors did not differ significantly regarding mental well-being and perceived preoperative hospital and surgery-related stress (Krampe et al., 2020). In fact, the word “tumor,” whether malignant or benign, can be psychologically stressful for patients. In this regard, effective counseling and patient education would be helpful in alleviating psychiatric distress. However, patients with malignant sinonasal tumors probably need postoperative radiotherapy and/or chemotherapy, which may cause worse posttreatment QOL when compared to patients with benign sinonasal tumors (Deckard et al., 2015).

In our study, we found that the overall symptom scores, facial pain/pressure scores, postnasal drip scores, and SNOT-22 scores were positively correlated with the scores of psychometric tests, indicating that patients at risk of psychiatric disorders have higher sinonasal symptom burden or that patients experiencing higher sinonasal symptom burden are more likely to be affected by psychiatric disorders. Considering that the SNOT-22 score was most closely correlated with scores of psychometric tests, and the SNOT-22 scale is routinely completed by patients in the rhinology practice, we further proposed that patients with an SNOT-22 score of more than 18 should be evaluated for the risk of psychiatric disorders and referred to a psychiatrist when necessary.

One of the limitations of our study is the small sample size and the heterogeneous histologic types of sinonasal tumors, which may limit the generalizability of our results. The incidence of sinonasal tumors is very low, and a multi-institutional study should overcome the problem of a small sample size in the future. Another limitation of our study is that the cross-sectional design could not determine the direction of causality between the symptom burden and psychiatric disorders. A well-designed longitudinal study would help to clarify causality.

In conclusion, depression, anxiety, insomnia, and SSD are prevalent in patients with sinonasal tumors but likely underdiagnosed. Regardless of whether the tumor is malignant or benign, the otolaryngologist should have a low threshold to ask the patient about psychiatric symptoms. The SNOT-22 score is closely associated with scores of psychometric tests and could be used to identify patients with sinonasal tumors at risk of psychiatric disorders (SNOT-22 > 18). Given the limited sample size, the results of our study should be interpreted with caution. A multi-institutional study with a large sample size is warranted in the future.

## Data Availability

The raw data supporting the conclusions of this article will be made available by the authors, without undue reservation.
